# Trichostatin A Promotes the Conversion of Astrocytes to Oligodendrocyte Progenitors in a Defined Culture Medium

**Published:** 2019

**Authors:** Leila Zare, Hossein Baharvand, Mohammad Javan

**Affiliations:** a *Department of Physiology, Faculty of Medical Sciences, Tarbiat Modares University, Tehran, Iran.*; b *Department of Stem Cells and Developmental Biology at Cell Science Research Center, Royan Institute for Stem Cell Biology and Technology, ACECR, Tehran, Iran.*; c *Department of Developmental Biology, University of Science and Culture, ACECR, Tehran, Iran.*; d *Department of Brain and Cognitive Sciences Cell Science Research Center, Royan Institute for Stem Cell Biology and Technology, ACECR, Tehran, Iran.*

**Keywords:** Human astrocytes, Oligodendrocyte progenitors, Trichostatin A, Cell fate conversion, Myelin repair, Multiple sclerosis

## Abstract

The generation of oligodendrocyte progenitor cells (OPCs) offers tremendous opportunities for cell replacement therapy in demyelinating diseases such as multiple sclerosis (MS) and spinal cord injury. Recently, the prospect of reprogramming terminally differentiated adult cells towards another mature somatic cell or progenitor cells without an intermediate pluripotent state has been of interest. Trichostatin A is a histone deacetylase inhibitor which opens the chromatin and facilitates the transcription of silence genes. In this study, we have treated human astrocytes line U87 and primary culture of mouse astrocytes with TSA for 12 h, prior their transfer to OPC induction medium. Then we evaluated the morphology and the fate of the treated astrocytes at post-treatment days. Both cell lines acquired OPC morphology and expressed OPC specific markers. Following transfer to differentiation medium, U87-derived iOPCs differentiated to oligodendrocyte like cells and expressed PLP as a mature oligodendrocyte marker. Our results introduced TSA as an inducer for production of OPCs from astrocytes and could be considered a potential way for the treatment of demyelinating diseases.

## Introduction

Considering the pivotal role of histone deacetylases (HDACs) in chromatin remodeling and epigenetics regulation, their inhibitors have been in the focus of research in recent years. These sorts of chemicals are attractive as a new source of anticancer drugs e.g. breast cancer (1). Trichostatin A is a potent *in-vivo* and *in-vitro* inhibitor of HDACs (2) and is supposed to exert synergistic effects on some anti-tumor drugs and a dual anti-HDAC/Wnt mechanism seems to be involved (1, 3, 4).

Multiple sclerosis (MS) usually begins in early adulthood with an autoimmune inflammatory impact on oligodendrocyte cells or the myelin sheath. Symptoms of the disease include movement disorders, sensory disturbances and cognitive and visual deficits (5-7).

Evidence indicates that the relapsing-remitting multiple sclerosis, which is characterized by distinct attacks followed by remission, may be mediated by an autoimmune reaction (8). 

The subsequent chronic progressive phase of disease is due to long lasting demyelination which leads to degeneration of the underlying axon (9). Therefore, production of oligodendrocyte progenitors (OPCs) for cell replacement therapy seems to be of special interest for repairing the demyelinated axons within the plaques and preventing them from subsequent axon degeneration.Recently, the direct conversion of terminally differentiated somatic cells to other mature or progenitor cells without an intermediate pluripotent state has become attractive due to lower risk of tumorigenicity (10-13). 

Direct conversion of astrocytes into neurons using overexpression of the neurogenic transcription factors in presence of small molecules has been reported (14-20). In our previous work we showed direct conversion of astrocytes into neuroblasts by miR-302/367, both *in-vivo* and *in-vitro*. 

The induced neuroblasts were capable to differentiate to mature neurons *in-vitro*, and produce action potentials in response to electrical stimulation (21). 

 In our previous works we have showed that in the presence of a demyelination niche, miR-302/367 overexpression converted the astrocytes into oligodendrocyte-like cells (22). We also indicated that overexpression of SOX10 results in transdifferentiation of astrocytes to oligodendrocyte lineage cells (23). 

Transcription factor-mediated reprogramming is mostly carried out via viral infection. A safe alternative approach would be development of chemical induced approaches to convert astroglial cells desired into desired cells using small molecules. 

As examples, fibroblast cells have been chemically converted to iPSCs (24, 25), hepatocytes (26), cardiomyocytes (27), NSCs (28, 29), and even neuronal cells (19, 30). In our recent report we converted astrocytes to oligodendrocyte lineage cells using chemicals both *in-vivo *and *in-vitro *in 1321N1 cell line (31). In current study we aim to reprogram cultures astrocytes line U87to induced OPCs (iOPCs) by exposure to TSA, followed by investigating the ability of iOPCs to differentiate to myelinating oligodendrocytes in culture. 

## Experimental

*Astrocyte culture and Characterization*

Human astrocytes line U87 were cultured in Dulbecco›s Modified Eagle›s medium (DMEM: Invitrogen), fetal calf serum (FCS: 10%; Invitrogen) and penicillin/streptomycin (1%; Invitrogen). The culture medium was changed every two days. 

For preparing primary astrocyte culture, mouse cortical astrocytes were extracted from P1 to P4 mouse pups based on the protocol introduced by Schildge *et al*. (32). Briefly, after sampling the cortices under sterile conditions, meningeal tissue were removed carefully, then the cortices were dissected into small pieces. After washing and trypsinization, the debris were removed. The tissue was disassociate to single cells using vigorous pipetting in astrocyte medium. The isolated cells were suspended in astrocyte media and transferred to T75 tissue culture flask coated with poly-D-lysine (Sigma-Aldrich). After 7 days, the confluent culture was shaked at 240 rpm for 6 h followed by vigorous shake by hand for 1 min to remove microglia and OPCs. Remained astrocytes were washed and passaged to a new T75 tissue culture flask and incubated. The medium was changed every 2 to 3 days. Two weeks after the seeding, astrocytes were entered into the experiment. The yield of astrocyte was around 1.5 million cells per 4 extracted mice. 

*Astrocyte treatment and induction*

The astrocytes were treated with TSA (10 nM, Sigma-Aldrich) for 12 h. Then the treated cells were seeded in OPC inducing medium including DMEM-F12 (Invitrogen), Platelet-Derived Growth Factor (PDGF: 20 ng/mL; RoyanBiotech), Fibroblast Growth Factor (FGF: 10 ng/mL; RoyanBiotech), Epithelium Growth Factor (EGF: 10 ng/mL; RoyanBiotech), Non-Essential Amino Acids (NEAA: Gibco), Smoothened Agonist (SAG: 1μM; Calbiochem), penicillin/ streptomycin (1%; Invitrogen), and B27 and N2 supplement (1X; Invitrogen). When required, for differentiation induction, triiodothyronine (T3: 40 ng/mL; Sigma-Aldrich) was added to the basic OPC media and OPC mitogens (EGF, PDGF, FGF) were removed.

*Immunostaining*

Both starting astrocytes and produced cells were examined using immunocytofluorescence studies against specific markers. Cultured cells were washed three times with PBS, fixed with 4% paraformaldehyde for 10 min at room temperature. After extensive washing with PBS for three times, cells were permeabilized with 0.2% Triton X-100 for 20 min, then blocked in 10% normal goat serum for 1 h. The appropriate concentrations of primary antibodies were added and cells were kept at 4 °C, overnight. Then cells were washed for three times with PBS. After the addition of secondary antibody and incubation for another 30 min, the cells were extensively washed and coverslipped using a DAPI containing mounting medium. The slides were investigated under fluorescence microscope and photographed using a DP-72 camera for offline analysis. Table 1 shows information for all primary and secondary antibodies.

## Results

*Characterization and induction of human astrocytes*

**Figure 1:**
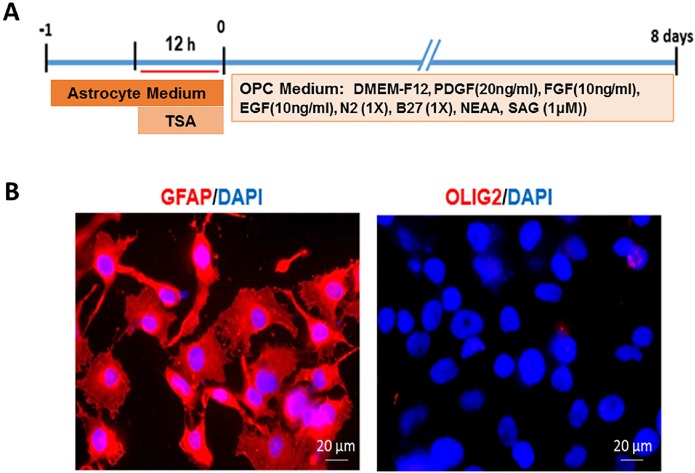
Interventions performed on human astrocytes line U87 to produce induced oligodendrocyte progenitors and characterization of starting cells. A) The timeline of treatment and induction of U87 astrocytes. B) Expression of GFAP as an astrocyte marker by all cultured astrocytes and the lack of expression of Olig2 as an oligodendrocyte lineage marker showed the purity of starting cell. Scale bar: 20 μm.

Astrocytes, line U87, were used as starting cells. Following 12 h treatment with TSA, the astrocytes were transferred to OPC induction medium (Figure 1A). In order to characterize the starting cells for their purity, human astrocytes were stained with astrocyte specific marker GFAP. Results showed that astrocyte culture was pure (GFAP+), without contamination to oligodendrocyte lineage cells as determined by the lack of Olig2 + cells in the culture (Figure. 1B).

**Table 1:** The characteristics of antibodies which were used in immunocytofluorescence studies

**Target molecule**	**Species isotype**	**Label**	**Company**	**Final concentration**
GFAP	Rabbit polyclonal IgG	-	Dako, Z0334	1:300
Olig2	Rabbit polyclonal IgG	-	Abcam, Inc. ab9610	1:200
PLP	Rabbit polyclonal IgG		Abcam, Inc. ab28468	1:100
PDGF	Rabbit polyclonal IgG	-	SC-338	1:100
O4	Mouse monoclonal IgM	-	R &D Systems, MAB1326	1:200
Rabbit IgG	Goat anti-rabbit	Alexa Fluor® 488	Life Technologies, A11008	1:1000
Rabbit IgG	Goat anti-rabbit	Alexa Fluor® 568	Life Technologies, A11036	1:1000
Mouse IgM	Goat anti-mouse	Alexa Fluor® 568	Life Technologies, A21043	1:1000
Mouse IgM	Goat anti-mouse	Alexa Fluor® 488	Life Technologies, A21042	1:1000

**Figure 2:**
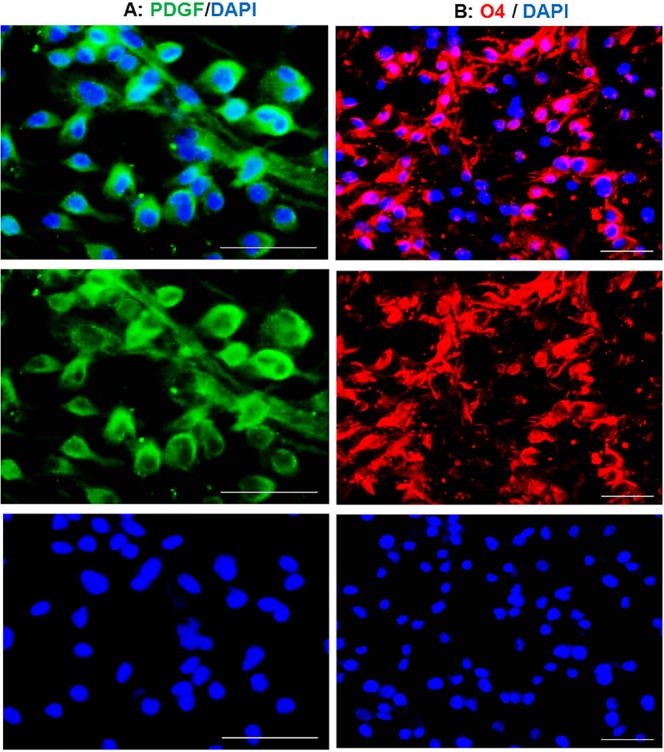
Conversion of the fate of TSA-treated human astrocytes to oligodendrocyte progenitors. The induced cells expressed PDGFR

(A) and O4 as oligodendrocyte progenitor markers at 8 days post induction. Scale bar: 50 μm.

**Figure 3:**
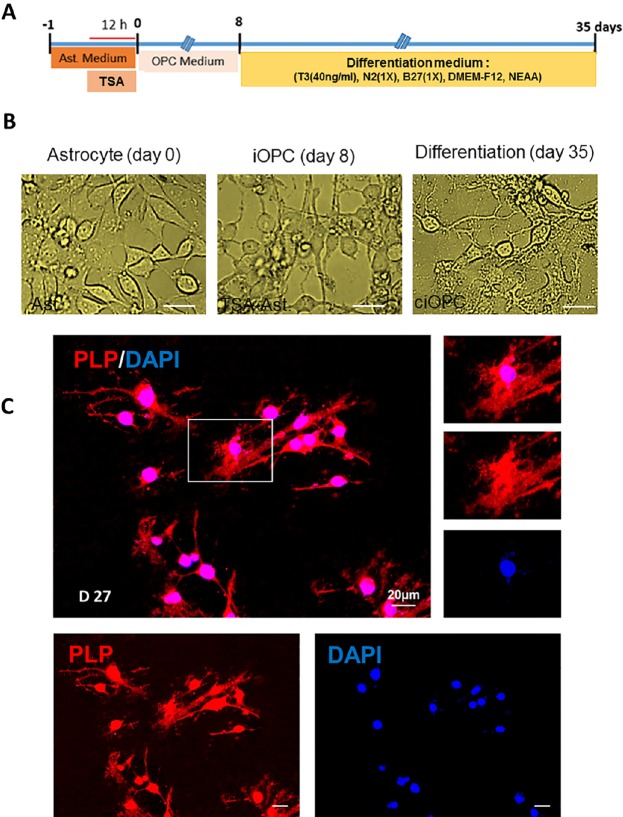
Differentiation of induced oligodendrocyte progenitor cells to mature oligodendrocytes. A) The timeline and the content of differentiation medium. B) Morphological changes in treated astrocytes during induction to oligodendrocyte progenitors and differentiation to oligodendrocytes. C) Characterizing the fate of differentiated astrocytes at 27 days post transfer into differentiation medium using immunostaining against myelinating oligodendrocyte marker PLP. Scale bar: 20 μm

**Figure 4:**
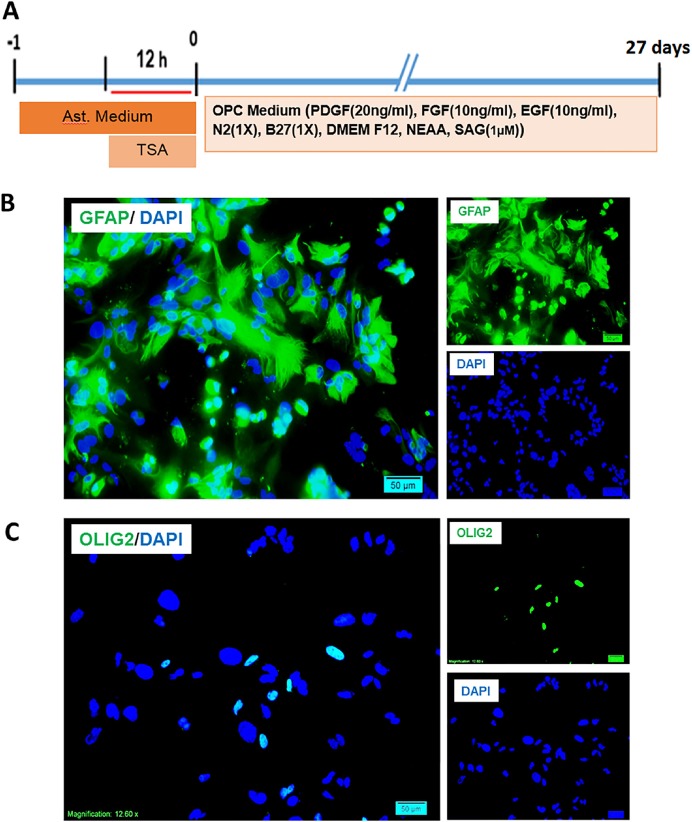
Interventions performed on mouse astrocytes for production of induced oligodendrocyte progenitors and characterization of starting cells. A) The timeline of treatment and induction of primary cultures of muse astrocytes and the content of induction medium. B) Expression of GFAP as an astrocyte marker by the cultured astrocytes and the low percentage of expression of Olig2 as an oligodendrocyte lineage marker. Scale bar: 20 μm

**Figure 5:**
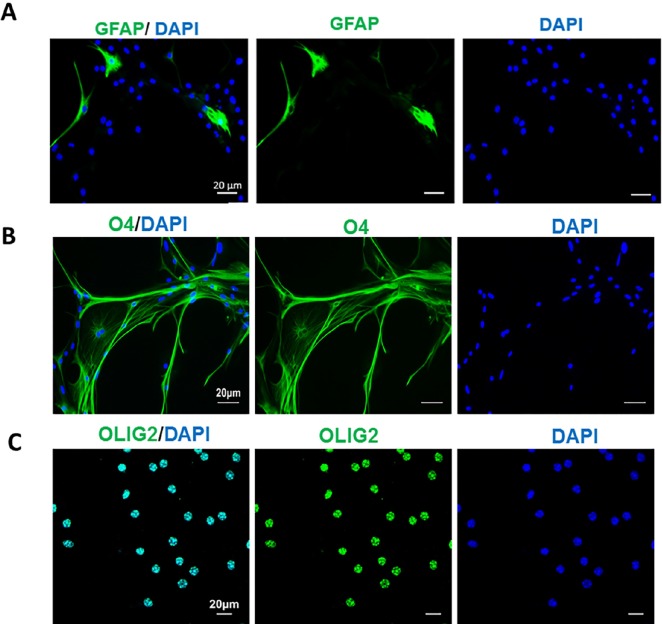
Conversion the fate of TSA-treated mouse astrocytes to oligodendrocyte progenitors. The induced cells were nearly negative for GFAP as an astrocyte marker (A) but expressed O4 (B) and Olig2 (C) as oligodendrocyte progenitor markers at 27 days post induction. Scale bar: 50 μm

*Conversion of human astrocytes to OPCs*

Eight days after transferring of TSA treated cells to OPC medium, OPC-like morphologies were observed in the culture. These induced cells were stained against PDGFR as a marker of OPCs. The main population of induced cells was positive for PDGFR (Figure 2A) which imply for the conversion of treated astrocytes to OPCs. This conversion was also confirmed by staining against O4, expressed in late OPCs. Overall estimation showed that about 90 percent of the cells were positive for O4 at day 8 post-induction (Figure 2B). Blue staining using DAPI shows the nuclei.

*Differentiation of human-iOPCs to oligodendrocyte-cells*

To confirm the OPC fate of induced cells (iOPCs), cells were transferred into the differentiation medium and kept for 27 days in presence of T3, while the mitogens were removed (Figure. 3A). Figure 3B shows the changes in the morphology of TSA-treated cells at 8 and 35 days later. Following 27 days culture in differentiation medium, iOPCs changed their fate with extending their process and protruding some flatted process. As it is mentioned in Figure 3C, staining of differentiated cells against PLP as a marker of myelinating cells, showed that iOPCs were differentiated to myelinating cells. Our results showed that 12 h treatment with TSA, promoted the astrocyte for conversion to oligodendrocyte lineage cells which were myelinating following directed differentiation. Non TSA-treated cells do not produced OPCs in OPC medium and gradually disappeared via cell death.

*Characterization and induction of mouse primary astrocytes*

To conform the conversion of astrocytes to OPCs in non-tumoric cells, primary mouse astrocytes were treated with TSA for 12 h and then transferred to OPC medium and kept for 27 days (Figure 4A), until they mentioned OPC like morphology. The purity of cultured cells were checked by immunostaining against GFAP and Olig2. Around 95 percent of the cells in astrocyte culture were astrocytes. Few numbers of cells were positive for Olig2 (Figure 4B). DAPI was used to stain the nuclei in blue.

*Conversion of mouse astrocytes to OPCs*

Following 27 days culture of TSA-treated astrocytes in OPC induction medium and observing the OPC-like morphologies, the cells were stained against GFAP as astrocyte marker, and O4 and Olig2 as OPC markers. Few numbers of cells were GFAP+ (Figure 5A), which showed the main population of cell had changed their fate from astrocyte to other cell types. Staining using O4 showed that most of the cells obtained OPC fate and express its marker. Very few nuclei were negative for O4. Cells were also stained by Olig2 as a marker of oligodendrocyte lineage cells. The stained cells were mainly positive for Olig2 which confirmed that TSA treatment was capable to convert astrocytes to OPCs, in OPC induction medium.

## Discussion

In the present study, we successfully converted both human and mouse astrocytes into OPC-like cells using exposure to TSA for 12 h and then transferring the cells into OPC medium. 

The dose of TSA and its exposure duration were selected based on a previous study (33). We checked both 12 and 24 h as exposure times, but due to the relatively high level of cell death within the astrocytes which were exposed for 24 h, we considered 12 h as optimized exposure time. 

The chemical agent which we used in this study was known as an epigenetics modifier through the inhibition of histone deacetylase enzymes. TSA, an antifungal antibiotic produced by *Streptomyces hygroscopicus*, (34) is a class I and II mammalian histone deacetylase (HDAC) inhibitor and can alter gene expression by interfering with the removal of acetyl groups from histones (2, 35). This drug also affect some signaling pathways and enhances the effect of chemotherapeutic agents (3, 4). Therefore, a plausible question concerning the results of this study is the mechanism in which TSA contribute to astrocyte conversion to OPCs. 

Epigenetic and regulation of transcription, both are suggested to involve in small molecules mediated cell fate conversion. Here, we used small molecule TSA for 12 h and only prior to OPC induction by a defined medium. Therefore, during the cell fate conversion there was no TSA in the medium and the modification of signaling pathways and modified transcriptional activity seems not a plausible mechanism for the effect of TSA. Epigenetic mechanisms seem to be rational for the effect of TSA on astrocyte conversion to OPCs. Exposure of astrocytes to TSA at the beginning of the procedure may increase the acetylation level of OPC specific gene and facilitates their transcription during the induction by OPC defined medium. Majumder and colleagues using5-azacytidine and TSA induced the differentiation of astrocytes from neural precursor cells (33). In the other word while in their study TSA facilitated the differentiation of neural stem cells to astrocytes, in our study it facilitated the conversion of astrocytes to OPCs. It may be concluded that TSA do not induce a specific cell fate, but facilitates the cell fate conversion which seems to be due to its effect on the starting cell epigenetics. 

Pre-treatment of U87 astrocytes with TSA caused the conversion of astrocytes to iOPC within 8 days, while for mouse astrocyte primary culture, the conversion of TSA-treated astrocytes into OPCs took 27 days of induction with defined medium. Previous study by Anokye-Danso *et al*. showed that higher level of histone deacetylase activity persists in the mouse cells (36). This report may explain why mouse astrocyte required longer time for fate conversion following treating with TSA. 

iOPCs generated from U87 astrocytes were passaged in our experiments for 4-5 times. It seems that human iOPCs could proliferate enough to produced adequate cell numbers for therapeutic applications, when required. iOPCs prepared from muse cortical astrocytes also proliferated and made the culture dish confluent, but during the passage they mainly went under apoptosis; therefore, further optimization of their culture condition is required. In another experiment, Najm *et al*. reported that iOPCs generated from fibroblasts by Sox10, Olig2, and Zfp536 transcription factors were capable to be expanded up to 5 passages (37). Recently Kim *et al*. (38) reported that a single transcription factor Oct4 in combination with defined OPC induction medium was sufficient to generate iOPCs from the mouse fibroblast. Following 35 passages, their iOPCs were self-renewing with high efficiency in differentiation both *in-vitro* and *in-vivo*. 

While the induction of OPCs from neural stem cells is time consuming suing current available protocols, they can be differentiated into astrocytes more quickly. Our results may suggest production of OPCs through differentiation of neural stem cells to astrocytes as an alternative way. Site specific delivery of chemicals like TSA into the glial scars may provide another application for our results. Conversion of reactive astrocytes to OPCs provides a two-fold beneficial effect on the treatment of MS via conversion of reactive astrocytes which are inhibitory for myelin repair to OPCs which can participate into repair mechanisms. This strategy may work with other neural disorders such as spinal cord injury which is characterized with demyelination induced axonal degeneration in some parts of its pathology. 

## Conclusion

These results show that iOPC could be generated directly from adult human astrocytes using small molecule TSA as an epigenetic modulator. Then these cells were capable to differentiate into mature and myelinating oligodendrocytes, *in-vitro*. The data were confirmed by conversion of primary cultures of mouse astrocyte into iOPCs. This approach seems promising for converting glial scar reactive astrocytes or neural stem cells derived astrocytes into oligodendrocyte progenitor cells in a wide range of demyelinating diseases like MS. 
